# Maintenance Therapy with Aromatase Inhibitor in epithelial Ovarian Cancer (MATAO): study protocol of a randomized double-blinded placebo-controlled multi-center phase III Trial

**DOI:** 10.1186/s12885-022-09555-8

**Published:** 2022-05-06

**Authors:** Pamela M. J. McLaughlin, Maximilian Klar, Tibor A. Zwimpfer, Gilles Dutilh, Marcus Vetter, Christian Marth, Andreas du Bois, Carmen Schade-Brittinger, Alexander Reuss, Claudine Bommer, Christian Kurzeder, Viola Heinzelmann-Schwarz

**Affiliations:** 1 Department of Gynecology and Gynecologic Oncology, University Hospital Basel, Basel, Switzerland; 2 Department of Gynecology and Gynecologic Oncology, University Hospital Freiburg, Freiburg, Germany; 3 Department of Biomedicine, University and University Hospital Basel, Basel, Switzerland; 4 Department of Clinical Research, Clinical Trial Unit, University of Basel Hospital, Basel, Switzerland; 5 Department of Medical Oncology, Hematology and Immunotherapy, Cantonal Hospital Baselland, Medical University Hospital, Liestal, Switzerland; 6 Department of Gynecology and Gynecologic Oncology, University Hospital Innsbruck, Innsbruck, Austria; 7 KEM: Evangelische Kliniken Essen Mitte, Essen, Germany; 8 Coordinating Center for Clinical Trials, Philipps University Marburg, Wiesbaden, Germany

**Keywords:** Aromatase Inhibitor, Letrozole, Generic, Hormone therapy, Ovarian Cancer, Maintenance therapy, Quality of life, Randomized controlled phase III trial

## Abstract

**Background:**

A high percentage of epithelial ovarian cancers (EOC) express the estrogen receptor (ER), which is an ideal target for endocrine therapy. Letrozole is a proven, potent aromatase inhibitor, extensively tested and used in the treatment of ER positive breast cancer. In addition, it seems a potent drug for patients with heavily pre-treated OC as demonstrated in several distinctive settings. However, it has never been evaluated prospectively in a maintenance setting for ovarian cancer after standard of care. The here proposed trial aims to define a population of EOC patients, who would benefit from the effectiveness of the generic agent letrozole, with little expected toxicity and thus beneficial impact on overall quality of life (QoL).

**Methods:**

In this international multicenter randomized, placebo-controlled phase III trial at clinical centers in Switzerland, Germany and Austria, we plan to include 540 patients with primary, newly diagnosed FIGO Stage II to IV and histologically confirmed low- or high-grade serous or endometrioid epithelial ovarian/fallopian tube/peritoneal cancer. Patients are randomized in a 1:1 ratio into two groups: receiving blinded study treatment (letrozole or placebo tablets). When assuming a HR of 0.7, a median PFS of 18 months in the control arm and a median PFS of 25.7 months in the treatment arm, a two-sided alpha level of 5%, 3.5 years recruitment and 1.5 years observation time, we expect 330 events to have occurred within these 5 years in the total cohort yielding a power of 90%. Follow-up data for the whole cohort will be collected for up to 10 years and for the low-grade cancer for up to 12 years.

**Discussion:**

The here proposed randomized phase III trial aims to identify patients with EOC in the maintenance setting, who benefit from the effectiveness of the letrozole, by proving its efficacy whilst maintaining a high standard of QoL due to the limited toxicity expected in comparison to the current alternative drugs on the market for this treatment phase.

**Trial registration:**

This trial is registered at clinicaltrials.gov under the identifier NCT04111978. Registered 02 October 2019.

**Supplementary Information:**

The online version contains supplementary material available at 10.1186/s12885-022-09555-8.

## Background

The prognosis of patients with ovarian cancer is still poor with a 5-year relapse rate of 75% for advanced high grade serous and endometrioid ovarian cancers (HGOC) [1–3]. However, better treatment options including maximal cytoreductive surgical efforts and new targeted-therapies has improved the outcome of particularly HGOC over the last decade. Several drugs have been evaluated in clinical trials in the primary and recurrent setting. The most promising medical strategies to delay progression of ovarian cancer after primary surgical and adjuvant treatment as of today are (a) PARP-inhibition with olaparib [4, 5], niraparib [1], rucaparib [6, 7] and others or (b) inhibition of angiogenesis with targeting agents like bevacizumab [8–10], pazopanib [11], cediranib [12, 13], trebananib [14] and others. Bevacizumab, a vascular endothelial growth factor (VEGF)-antibody is approved for maintenance treatment based on a post-hoc analysis for high-risk cancers [15]. Olaparib, a PARP-inhibitor, is approved for high-grade serous ovarian cancer in the recurrent setting after re-introduction of platinum-based chemotherapy [15]. Additionally, Olaparib showed tremendous benefit in BRCA mutated patients in the first adjuvant SOLO-1 maintenance trial [4]

Recently, the results of three separately conducted clinical trials with various PARP inhibitors and different combination treatments, PRIMA, PAOLA, and, by Coleman et al*.,* 2019, VELIA, demonstrated substantial benefit when incorporating PARPi upfront in the treatment of newly diagnosed ovarian cancer, leading to a meaningful increase of progression-free survival [1, 5, 16]. However, with a substantial treatment discontinuation (12%-54%), dose reduction (28%-70.9%), and dose interruption (20%-79.5) due to adverse events [1, 4, 5], and with a note that, the impact of improved surgical resection techniques could not be ignored as residual disease, after upfront surgery, is still the best prognostic indicator for relapse [3].

Despite all these new treatment options and the subsequent increase on progression-free survival (PFS) for distinct groups of ovarian cancer patients, the overall survival (OS) remains poor with a high relapse rate [1, 2, 4]. This underlines the need for the exploration of treatment options that will increase PFS and OS without decreasing quality of life (QoL).

### Rationale

Anti-hormonal therapy is an old, but important treatment option with recent new and promising results on the maintenance phase of patients treated for ovarian cancer until Time to First Subsequent Treatment (TFST) [17, 18]. For early and advanced estrogen receptor (ER) positive breast cancer, endocrine therapy including maintenance phase therapy is the gold standard [19–21]. The most active drugs are tamoxifen, aromatase inhibitors and fulvestrant, which have considerably improved the prognosis for women with breast cancer [22].

Aromatase inhibitors (AIs) inhibit the estrogen production in postmenopausal women by more than 90%. In the adjuvant setting of breast cancer patients, the therapeutic effect of AIs has shown to be superior to that of tamoxifen [23].

Expression of aromatase mRNA and protein have been found in 33–81% of ovarian cancers [19]. Preclinical data has shown that the growth of ovarian cancer cells is prohibited *in vivo* and *in vitro* by the endocrine therapy against ER positive OVCAR-3 HGOC cells [22]. In addition, *in vitro* studies show an anti-tumor effect of AI on ovarian cancer cells, which was associated with aromatase activity and ER expression [24].

Several small series suggested a benefit in low-grade serous ovarian cancer (LGOC) patients, suggesting that there are subgroups of patients with a specific tumor biology that respond very well to endocrine therapy [25, 26]. LGOC is a rare histological subtype and biologically distinct from HGOC. Only approximately 5–10% of all ovarian cancers are of low-grade serous type. Gershenson et al*.* [17, 18]*,* presented retrospective analyses of endocrine maintenance therapy (with diverse regimens, including anastrozole, letrozole and tamoxifen) for LGOC. For patients receiving endocrine maintenance therapy (*n* = 70) after platinum-based adjuvant chemotherapy, PFS significantly improved as compared to patients under observation only (*n* = 133, 64.9 months; 95% CI 43.5 to 86.3; versus 26.4 months; 95% CI 21.8 to 31.0, *p* < 0.001) [17]. Most patients received treatment with letrozole (54%) or tamoxifen (28%) [13]. In 2012, the same authors described already a high clinical benefit rate in a retrospective analysis obtained from medical records ranging from 1989 until 2009 covering 64 patients with recurrent LGOC [18]. In spite of the known limitation of retrospective analyzed data as a long study period, incomplete data, potential referral bias, heterogeneous therapies, and varying follow-up practice patterns, patients receiving different regimens of endocrine maintenance therapy for relapse showed a response rate of 9% and stable disease of 62% [18]. This is of major significance as—similar to ER-positive breast cancer patients – LGOC affects mostly younger women with a rather poor prognosis [17].

The rationale for exploring endocrine therapy for the treatment of ovarian cancer patients is based on the high ER/PR expression as a predictive marker, since ovarian cancer is partly driven by the estrogen-pathway [27].

In spite of these clear distinctions between HGOC and LGOC, primary treatment strategies in both diseases have so far been similar, i.e. maximal surgical cytoreduction, followed by adjuvant chemotherapy with carboplatin and paclitaxel. None of the studies and analyses on endocrine treatment of ovarian cancer so far were prospective, in the maintenance setting, and potentially in combination with other maintenance treatments, but rather as a stand-alone treatment regimen in heavily pre-treated patients. Of note, generally no information in regards to ER/PR expression was provided.

As a pilot, we performed a small single-site prospective observation trial in 50 HGOC FIGO III/IV patients and analyzed the results together with three other patient cohorts. We found a marked improved PFS at 24 months: 60% when taking Letrozole versus 38.5% in the control group (*p* = 0.035). This effect was even more present in patients treated additionally with Bevacizumab; 20.8% of patients had no recurrence after 12 months compared to 87.5% when taking Letrozole in addition to Bevacizumab (*p* = 0.026). This positive effect was particularly evident when the treatment was initiated within three months after the end of adjuvant chemotherapy [28].

It is remarkable that endocrine treatment has only been used in the relapsed setting of ovarian cancer and that its role in primary or maintenance treatment has not been studied. Although, for more than 40 years, tamoxifen and later aromatase inhibitors have been studied in smaller cohorts and studies. A literature review covering over 50 trials including retrospective analysis, demonstrated a clinical benefit rate ranging from 0–95% when focusing on the most commonly used drugs, tamoxifen and aromatase inhibitors. These ambiguous results are probably caused by the heterogeneous patient’s characteristics and the fact that these patients received the treatment, mostly, during later lines [29].

In the relapsed setting there are little data comparing chemotherapy versus tamoxifen. One larger phase III trial in the platinum-resistant setting compares tamoxifen 40 mg/d with standard of care paclitaxel or liposomal doxorubicin. Patients on chemotherapy had longer PFS (12.7 vs. 8.3 weeks, HR, 1.54; 95% CI, 1.16–2.05; log-rank *p* = 0.003) but experienced more toxicity and poorer QoL [30]. As a result of these studies, endocrine therapy has currently been listed in the NCCN guidelines as an optional treatment strategy in the relapsed setting [31].

To our knowledge (on 14. December 2021 we searched clinicaltrials.gov and trialsearch for ongoing maintenance trials with Letrozole, using the search terms ‘ovarian cancer’, ‘ER’, ‘endocrine therapy’ and ‘letrozole’, identifying one other trial (NCT04095364) for ovarian cancer patients), there is only one other clinical trial evaluating aromatase inhibitors as a first line monotherapy and in the maintenance setting of LGOC (NCT04095364) whereas it has been commonly evaluated in the relapsed setting [29]. Furthermore, there are no QoL data of aromatase inhibitor after 1^st^ line treatment in the maintenance setting. To this end, the MATAO trial evaluates letrozole versus placebo in the maintenance setting in low and high grade serous and endometrioid ovarian cancer patients accompanied by assessment of the quality of life questionnaires EQ-5D-5L, FACT-ES, and FACT-O in combination with the CD-RISC-10 resilience, G8 fragility and ACCI comorbidity scores.

### Aims

There is a need to evaluate endocrine therapy in a prospective large phase III trial including and focusing on QoL aspects. We present a phase III randomized double-blind placebo-controlled multi-center trial that will assess the effect of the aromatase inhibitor letrozole as maintenance therapy in patients with FIGO Stage II-IV low and high-grade ovarian cancer of endometrioid or serous histotype. This experimental arm will be compared to the current standard, namely use of no maintenance endocrine therapy after the end of adjuvant chemotherapy, whereas, combined treatment with the new approved treatment options in this phase is allowed and has been proven safe [32, 33].

Based on the evidence presented above, we hypothesize that the letrozole maintenance arm will lead to a prolongation of PFS in these patients with no decrease of QoL. PFS has been selected based on previous data from our own study [28] and a recent meta-analysis [34] demonstrating a high potential benefit in regards to prolongation of the time to first relapse by 8 months using endocrine treatment in the maintenance setting.

Moreover, this study aims to identify the population that would benefit the most from this endocrine therapy in respect to prolonged quality of life (QoL). QoL will be assessed, prior, during, and after the intervention period, by both validated QoL questionnaires as well as by activity measurements. In addition, the survival status will be evaluated in relation to fragility and vulnerability as well. To this end, the G8 score will be used to assess fragility by patients over 75 years of age and the CD-RISC-10 score to assess resilience in the whole cohort.

## Methods/design

### Study design and sites

**Fig. 1:**
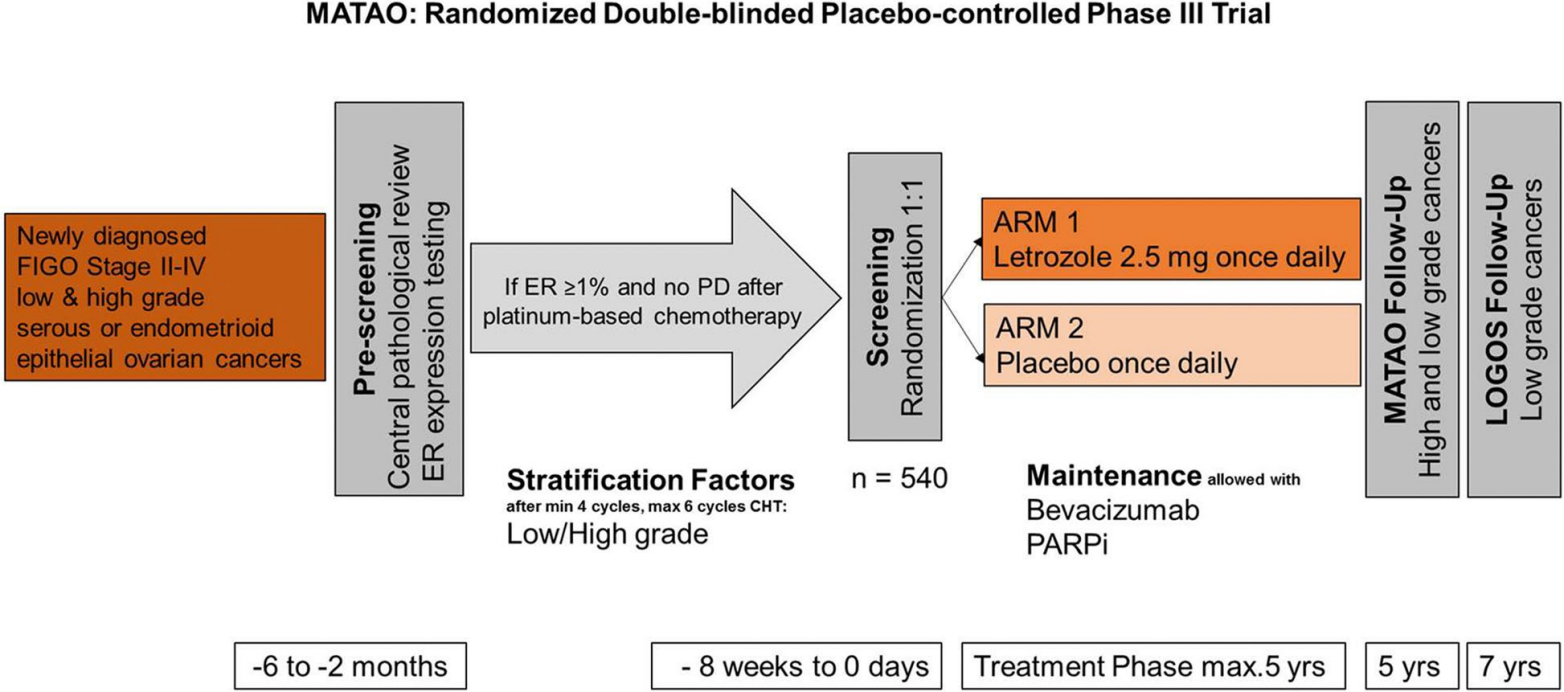
Schematic representation of MATAO study design, covering the maximum time schedule per patient depending on the differences in in histopathological grading. *ER*   Estrogen Receptor*, PARPi*   Poly(ADP-Ribose)-Polymerase 1 inhibitor

This study is designed as a parallel group, multi-center, superiority, randomized, placebo-controlled, phase III Trial within the ENGOT research network including 21 Swiss, 30 German and 8 Austrian Gynecological Cancer Centers (Fig. 1).

### Patient recruitment and screening

Potential participants are being identified during tumor board by their treating oncologist. Patients already undergoing chemotherapy are also potential study candidates, if use of the scores are routinely in place.

If the ER expression is positive and the pathology review confirmatory for both low or high-grade serous or endometrioid ovarian cancer, including fallopian tube and primary peritoneal cancer, patients undergo a second screening process after subsequent treatment with chemotherapy to ensure that the tumor has not progressed under chemotherapy. Written informed consent is obtained from each patient prior to randomization.

### Inclusion criteria

- Patients must be ≥ 18 years of age
- Willing and able to attend the visits and to understand all study-related procedures
- Primary, newly diagnosed FIGO Stage II to IV and histologically confirmed low or high grade serous or endometrioid epithelial ovarian/fallopian tube/peritoneal cancer
- (Interval-) debulking performed
- Eastern Cooperative Oncology Group (ECOG)-Performance Status 0–2
- Signed informed consents (ICF-1; ICF-2)
- Paraffin-embedded tissue or paraffin-embedded cell block (from ascites) available
- Positivity (≥ 1%) for ER expression (as determined by the central Histopathology Core Facility of the MATAO trial)
- At least 4 cycles of platinum-based chemotherapy (neoadjuvant allowed)
- Negative serum pregnancy test in women of childbearing potential (women of childbearing potential defined as: premenopausal or less than 12 months of postmenopausal amenorrhea, and who have not undergone surgical or radiation sterilization)

Note: Patients under concurrent maintenance treatment with Bevacizumab and/or PARP Inhibitors are eligible

### Exclusion criteria

- Progressive disease at the end of adjuvant treatment
- Any other malignancy within the last 5 years which has impact on the prognosis of the patient
- Less than 4 cycles of chemotherapy in total
- Contraindications to endocrine therapy
- Inability or unwillingness to swallow tablets
- Women of childbearing potential (not having had nor will getting a surgical resection, prior to the intervention in the therapeutically maintenance setting)
- Pregnant or lactating women
- Patients with a known intolerance to galactose, lactase deficiency and glucose-galactose mal-absorption

### Randomization

After written informed consent has been obtained and all eligibility information has been entered into the system and all eligibility criteria are met, the secuTrial® database will provide the study site with a unique, anonymous patient identifier (randomization number). Subjects will be assigned 1:1 to letrozole or placebo. Randomization is performed from the secuTrial® system by stratifying the grade of cell differentiation (low or high) using the variance minimization procedure. After full completion of a subject’s enrollment the secuTrial® system creates automatically a confirmation of the successful randomization process, sent via Email blinded to the individual site and the coordinating office, and unblinded to the Clinical Manufacturing Organization (CMO).

### Blinding procedures

Physicians, study nurses, patients, outcome assessors, involved pharmacists and statisticians will be blinded to the allocated treatment. Letrozole and the placebo will be visually indistinguishable and will be provided in the same packed manner by the CMO. Neither the investigator/study team nor the patient will know what they are receiving, and it shall remain so until primary statistical analysis was performed. The CMO, who is not directly involved in the study, will label, pack and dispense the medication according to the results of the randomization procedure. Both, trial treatment and placebo are indistinguishable, apart from medication ID on the package, of which only the distributor and a dedicated person of the CRO have information.

This level of blinding is maintained throughout the trial until the primary analysis of the whole cohort. The final PFS analysis of the whole cohort in the study design of MATAO is scheduled, minimum 1.5 years after inclusion of the last patient, this will take place maximum after 5 years and 3 months. However, recruitment of LGOC patients need to be extended for another 2 years to meet the required statistical number of patients. This yields an interim analysis of the low-grade sub-group. The clinical project coordinating management, the patients and the site’s PI remain blinded for the low-grade group for the whole conduct of the trial.

### Intervention

Patients assigned by randomization to ARM1 (experimental arm) receive letrozole 2.5 mg (Femara®, Novartis) and patients assigned to ARM2 (control arm) will receive placebo (Novartis). The start is within 14 days after randomization from initial visit (M0), and orally taken once daily for a maximal total duration of 5 years or until symptomatic relapse, or other discontinuation reasons.

The tablet can be taken with or without food and should be swallowed whole with a glass of water or another liquid.

Letrozole or Placebo will be dispensed by the study nurse to the patient during her consultation at the center. The patient will receive 3-monthly supply of the experimental drug (after 2 years. 6-monthly supply). The study visits are scheduled according to the usual routine visits during the active study period.

### Objectives

The primary objective in this study is to evaluate the effect of letrozole maintenance therapy after standard surgical and chemotherapy treatment on PFS compared to placebo in patients with newly diagnosed ER positive epithelial ovarian cancer (histologic subtype: serous or endometrioid of low/high grade, including fallopian tube and primary peritoneal cancer). At FIGO Stage II-IV, with or without residual disease and with or without concomitant anti-VEGF and/or PARPi medication, whose cancer has not progressed by the end of adjuvant chemotherapy treatment.

The secondary objectives are to evaluate the letrozole maintenance treatment compared to placebo in terms of.

**Fig. 2:**
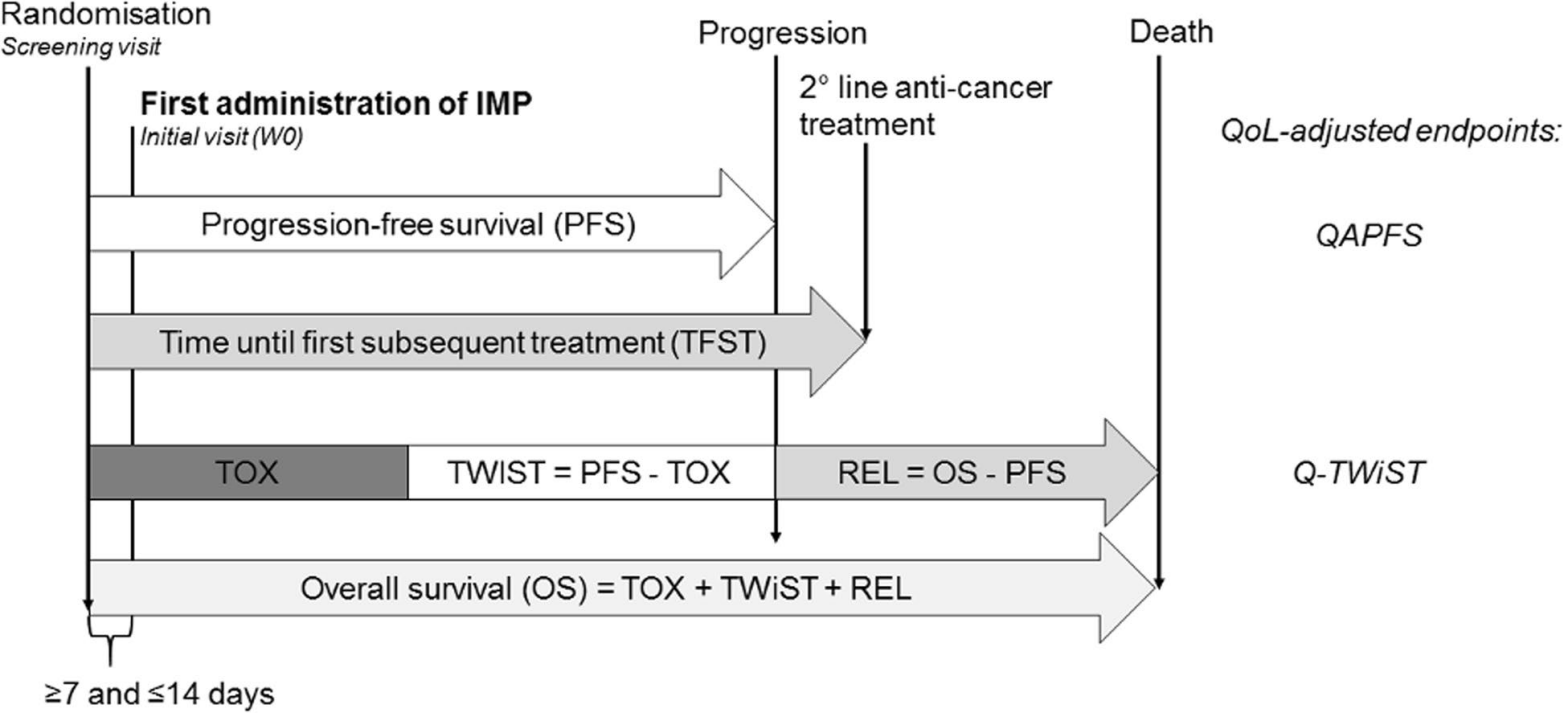
Overview of all time-dependent endpoints. *QAPFS   *Quality-adjusted Progression Free Survival, *Q-TWiST   *Quality-adjusted Time Without Symptoms and Toxicity,* TOX*   Toxicity*, TWIST*   Time Without Symptoms and Toxicity*, REL*  Relapse*, PFS   *Progression Free Survival*, OS   *Overall Survival

- Overall Survival (OS)
- Quality-Adjusted Progression Free Survival (QAPFS)
- Time to First Subsequent Treatment (TFST)
- Quality-adjusted Time Without Symptoms of disease and Toxicity (Q-TWiST)
- Health Related Quality of Life (QoL) assessed by EQ-5D-5L, FACT-ES and FACT-O (Fig. 2)

Additionally, the following explorative and translational objectives are evaluated:

- To explore the correlation between the activity values of the wearable activity tracker (objective QoL measuring tool) and the patient reported QoL scores
- To explore the feasibility of digitally obtained biomarkers for clinical trials
- To explore the efficacy of letrozole maintenance therapy in correlation to anti-VEGF and PARPi maintenance therapy as measured by PFS
- To explore the efficacy of letrozole maintenance therapy in comparison to no maintenance therapy (placebo) according to presence/absence of residual tumor tissue as measured by PFS
- To explore the effect of resilience (Connor-Davidson Resilience Scale 10 score categories) on PFS and over time
- To explore the resectability in primary or interval debulking surgery in correlation to the Age Adjusted Charlson Comorbidity Index (AACCI) and the G8 fragility questionnaire score
- To assess the safety and tolerability of the letrozole group in comparison to the placebo control group and historic breast cancer cohort
- To determine molecular markers of ER + /ER- and its association with clinico-pathological parameters [35–38]
- To determine pathways involved in estrogen response (e.g. EIG121, ESR1/2 and PI3KCA) [39–41]
- To determine the mutational load of involved tumors
- To determine the predictive role of a glycomic signature in association with clinico-pathological parameters [42]
- To determine synergistic effects of combined maintenance therapies by state-of-the-art molecular methods [43, 44]

### Sample size consideration and statistical analyses

#### Sample size considerations of the whole cohort

We estimated how many events are needed to achieve a 90% power to detect a hazard ratio (HR) of 0.70 between the two treatment arms (hazard letrozole/hazard placebo, assuming an exponential distribution of PFS). From the ICON7 adjuvant trial [10], which investigated bevacizumab in the adjuvant maintenance setting, it is expected that PFS is approximately exponentially distributed and the median PFS under standard care is 18 months. The assumed HR of 0.70 would increase the median PFS in the treatment arm to 25.7 months.

Calculations were done assuming a two-sided alpha level of 5% and a log-rank test for comparison. The approximation described by Machin et al., (2009) resulted in a required sample size of 330 events, i.e., cases with progression over both study arms [45]. The recruitment will continue until this number of events is observed and the power of 90% has been achieved. When assuming, in addition to the aforementioned conditions, a uniform recruitment rate during three and a half years and a dropout rate of 10%, a total number of 540 patients would suffice to achieve the target number of 330 events five years after study start (calculated using the method by Lachin & Foulkes, 1986) [46].

#### Sample size considerations specific for the low grade cohort, LOGOS

Assumptions about efficacy for the power calculations are based on the retrospective analysis of Gershenson et al*.* [17]. We assume exponential PFS distributions, the analysis by a two-sided log-rank test with alpha level 0.05, and a power of 80%. With an accrual duration of 7 years and a follow-up phase of 5 years after accrual of the last patient, a dropout rate of 15%, and a median PFS of 36 months in the control arm and 60 months in the experimental arm (i.e. a hazard ratio of 0.6 between arms). Because the main protocol of the MATAO trial schedules its final PFS analysis at approximately 5 years after inclusion of the last MATAO patient (while recruitment into the LOGOS stratum will still be ongoing) and states grade as a factor for subgrouping, technically, this constitutes an interim analysis of the LOGOS data. To account for this interim analysis, we plan an O’Brien-Fleming group sequential design with one interim look and one final look. The sample size / power calculations were performed using ADDPLAN version 6.1. Accounting for 15% dropout, 186 patients (i.e. approximately 2.2 patients per month) should be recruited into LOGOS in order to have the 158 evaluable patients, necessary to achieve a power of 80% with this O’Brien-Fleming group sequential design. The boundaries for the group sequential design at the interim look will be based on the actual number of observed events and will be calculated using the alpha-spending function for an O’Brien-Fleming design. The final analysis will be performed after observation of 121 events.

### Statistical analyses

Patients will be analyzed according to the intention-to-treat principle, i.e. all patients will be analyzed according to the treatment group they were allocated to, regardless of adherence.

### Primary analysis

A Cox proportional hazards regression model will be implemented to study whether treatment influences PFS. In this model, we will stratify by grade of cell differentiation (HGOC or LGOC). The proportional hazards assumption will be checked by inspecting the scaled Schoenfeld residuals and formally tested by performing the Grambsch-Therneau test. No matter whether a violation of proportional hazards is detected, the hazard ratio (HR) with its 95% confidence interval will be presented as well as the p-value for that HR. If there is an indication of a non-proportional hazard rates, the restricted mean survival time (and its difference between arms) at t* = 5 years, and its confidence interval will be reported since they provide a more insightful statistic in such cases (following the recommendations of Royston and Mahesh [47]) Furthermore, Kaplan–Meier curves will be plotted by treatment arm.

### Secondary analysis

**Fig. 3:**
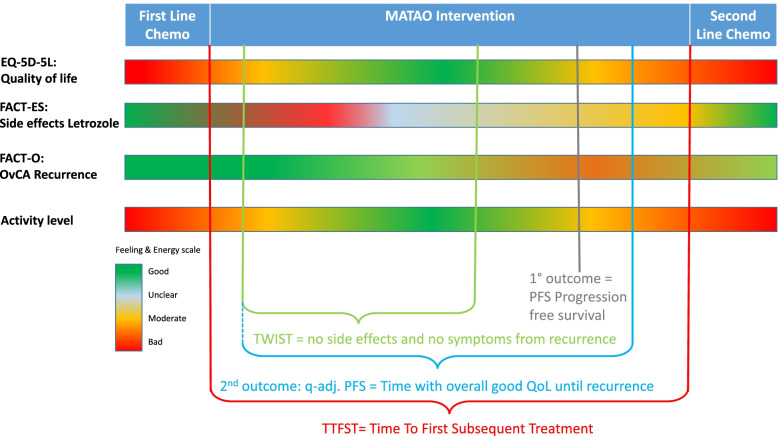
Envisioned results on a feeling and energy scale during maintenance phase, after at least 4 cycles of chemotherapy and potential surgery, determined via questionnaires and objectified via activity tracker. *EQ-5D*   European Quality of Life Scale*, *5-Dimensions*, FACT-ES*   Functional Assessment of Cancer Therapy—Endocrine subscale*, FACT-0*   Functional Assessment of Cancer Therapy – Ovarian cancer symptoms subscale*, PFS*   Progression Free Survival

The effects of the letrozole treatment on the secondary endpoints OS and TFST are tested applying the same analysis as described for the primary endpoint. The treatment’s effects on QoL, QAPFS and Q-TWiST are analyzed in a linear regression where baseline QoL is included as a covariate and the same stratum that was included for the primary analysis, i.e. grade of cell differentiation, is included as random effect. The sample size required for achieving the primary objective (assessing survival difference) will very likely suffice for a good estimate of the treatment arm difference for these continuous QoL outcomes. QAPFS, and Q-TWiST are based upon QoL assed using EQ-5D-5L and will be reported for each arm. In addition, descriptive statistics will be shown for all listed variables, broken down by study arm. The precise development of QoL over time will be presented graphically based on both the mean/median scores as well as on the proportion of patients reaching MICD, as will be described in detail in the subsequent statistical analysis plan. Similarly, the severity of side-effects of treatment over time will be shown (Fig. 3).

### Interim analysis

No interim analysis is planned for the primary analysis on the full set of patients (HGOC + LGOC). However, since the LGOC study recruitment continues after closure of the study for the whole cohort, the subgroup analysis of the LGOC should be regarded as an interim analysis by an O`Brien-Fleming group sequential design for this cohort. The boundaries for the group sequential design at the interim look will be based on the actual number of observed events and will be calculated using the alpha-spending function for an O’Brien-Fleming design. The final analysis will be performed after observation of 121 events.

### Subgroup analysis

We will explore the impact of the experimental treatment vs the control in the following subgroups:

- Bevacizumab treatment.
- PARP inhibitor treatment.
- Residual disease.
- Grade (LGOC/HGOC).
- Weak and strong ER expression.
- Resilience categories/scores.
- The elderly population (≥ 75 years).

For each subgroup, the primary analysis will be repeated. No other subgroups or subgroup analyses are pre-specified.

### Data management system and administration

The clinical trial data will be collected pseudonymized in an electronic data capture system, named secuTrial®. The secuTrial® database runs on a server maintained by the IT-Department of the University Hospital Basel. It is implemented (set-up and adjusted) by the data management group at the Clinical Trial Unit at the University Hospital Basel. Data management at the Clinical Trial Unit of the University Hospital of Basel will be performed as to their standard operating procedures, see CDMA Planning (2.0.1), CDMA Development, Testing and Release (2.0.1), CDMA Training (1.0.1), CDMA Locking and Closure (1.1.0) (SOPs CRO). Each study site is responsible for data entry into the secuTrial® database system.

In addition, the confirmed low-grade ovarian cancer data will be stored pseudonymized in the LOW-REG database created explicitly for the analysis of this sub cohort.

### Archiving and data retention

The secuTrial® database will be locked after all data has been monitored and all raised queries have been solved. Data will be exported and transferred to the Clinical Trial Unit University Hospital Basel according to internally defined processes (SOP’s CRO). Data will be archived by the Sponsor, except for the data and histological specimens of the subpopulation of low-grade ovarian cancers which are dispersed to the AGO (Germany).

### Monitoring

A monitoring team from the CRO will contact and visit all sites, either remote or physically, on initiation, during the study, and regularly if necessary. The Study Monitor will verify the adherence to the protocol and the completeness, consistence, and accuracy of the data being entered in the eCRF, to verify that the study is being conducted according to the protocol and within the specified period and the facilities and staff are adequately and trained, according to Risk Based Monitoring as described in the monitoring plan.

The Study Monitor will require access to all patient medical records including laboratory test results and surgery, pathology and radiology reports and supporting documents to verify the entries on the eCRF. The investigator (or his/her designee) should work with the Study Monitor to ensure that any problems detected during these visits are resolved and ensure that source data and documents are made accessible to the Study Monitor and answer questions by the Study Monitor.

### Participant`s confidentiality

The investigator will ensure that patient’s anonymity will be maintained during, as well as after the study (publication) and that their identities are protected from unauthorized parties. In eCRFs or additional trial documentation, patients will not be identified by their names, but by a unique identification code. The investigator must maintain documents with the patient’s identity at site hidden from the Sponsor in the ISF (e.g. patients written consent form in strict confidence at the site).

Direct access to source documents will be permitted for purposes of monitoring, audits and inspections. Monitors, auditors and inspectors will also maintain confidentiality of personal data of patients.

### Storage of biological material and related health data

The coded paraffin block from the central blinded pathology review and ER measurements (and the paraffin block provided after recurrence, if available) will be stored in the study biobank in the Histopathology Core Facility, Department of Biomedicine, University of Basel as described in the Swiss GO Trial Group biobank regulations. The paraffin blocks will be stored there for up to 20 years after the closing of this trial. Distinct written informed consent must be signed by each study participant for the analysis as well as the storage of these tumor tissues.

## Discussion

Despite optimized treatment for epithelial ovarian cancer the prognosis is poor with a maximum reported 5-year overall survival rate of up to 50% [1]. At present, the standard of care after a maximal surgical cytoreduction effort followed by adjuvant chemotherapy with carboplatin/paclitaxel is limited with maintenance therapy with bevacizumab for FIGO stages III-IV and additionally PARP inhibitor for HGOC with a BRCA mutation and partial or complete response to carboplatin/paclitaxel. Therefore, additional treatment options, particularly maintenance therapy regimens that might prolong this period of response are needed [48]. Nevertheless, some patients might be cured from the disease by the primary treatment already, so it is important that this treatment should be as tolerable as possible. A high percentage of epithelial ovarian cancer expresses ER, which is an ideal predictive marker for endocrine therapy response [49].

Letrozole is a potent aromatase inhibitor extensively tested and used in ER positive breast cancer patients and has also been tested in several smaller series for patients with heavily pre-treated epithelial ovarian cancer [29]. Letrozole in general is well tolerated and has been commonly used in breast cancer patients for more than 20 years. It has a preferable toxicity profile. Approximately 30% of patients have some mild symptoms like arthralgia, vaginal dryness and hot flushes. Recently, the combined use of Letrozole and Bevacizumab and Olaparib, respectively, has proven to be safe, as all toxicity observed was attributed solely to the new compounds and not to the letrozole [32, 33].

So far, it has not been prospectively evaluated in the adjuvant maintenance setting for ovarian cancer primary treatment. The MATAO trial aims to fill this gap and will evaluate for the first time prospectively the benefit of letrozole versus placebo in the maintenance setting of ER positive ovarian cancer.

Whilst long established in breast cancer, the highly effective option of aromatase inhibitors as adjuvant maintenance treatment has never been thoroughly examined for its use in ovarian cancer due to the disinterest of pharmacological industries. If this proposed trial would not be performed, in the near future every patient will be treated with drugs like PARP-inhibitors or bevacizumab as these will be the only drugs examined and approved for treatment in the maintenance setting of ovarian cancer [1, 4–6, 8, 9]. The toxicity profile of these drugs however, will not allow these to be applicable for all patients suffering from ovarian cancer, potentially excluding a large group of frail and vulnerable patients, the median age of onset being 63 years.

The proposed trial aims to create an opportunity for patients, who might benefit from the effectiveness of the generic agent Letrozole with proven efficacy and limited toxicity, with limited impact on their QoL whilst increasing their chance on prolonged PFS and ultimately on OS.

## Supplementary Information

**Additional file 1: Table 1.** Study schedule including procedure.

**Additional file 2: **Ethical committees & Study sites ENGOT-ov54/Swiss-GO-2/MATAO(LOGOS).

## Data Availability

The datasets that will be used and/or analyzed during the current study are not yet available as the study is presently ongoing, but will be available from the corresponding author in consultation with the national trial coordinators on reasonable request once recruitment and data collection are complete.

## Abbreviations

AACCI: Age Adjusted Charlson Comorbidity Index
ADAMON: Project to evaluate risk-adapted monitoring strategies for clinical trials
AE: Adverse Event
AESI: Adverse Event of Special Interest
AGO: Arbeitsgemeinschaft für Gynäkologische Onkologie/Working group Gynecologic Oncology
AI: Aromatase Inhibitor
ASCO: American Society of Clinical Oncology
AUC: Area under the curve
BASEC: Business Administration System for Ethical Committees
BRCA: Tumor suppressor gene for breast cancer
CA: Competent Authority (e.g. Swissmedic)
CA-125: Cancer Antigen 125
CDMA: Clinical Data Management Application
CEC: Competent Ethics Committee
CMO: Clinical Manufacturing Organization
CRA: Clinical Research Associate
CRF: Case Report Form
CRO: Contract Research Organization
CTCAE: Common Terminology Criteria for Adverse Events
CT: Computer Tomography
DSMB: Ata Safety Monitoring Board
ECOG status: Performance scale of Eastern Cooperative Oncology Group
eCRF: Electronic Case Report Form
EIG121: Estrogen-induced gene 121
EMA: European Medicines Agency
ENGOT: European Network for Gynecological Oncological Trial Groups
EOC: Epithelial ovarian cancer
EQ-5D-5L: European Quality of Life Scale, 5-Dimensions
ER: Estrogen Receptor
ESGO: European Society of Gynecological Oncology
FIGO: Fédération Internationale de Gynécologie et d'Obstétrique Gynécologie
FACT-ES: Functional Assessment of Cancer Therapy—Endocrine subscale
FACT-O: Functional Assessment of Cancer Therapy – Ovarian cancer symptoms subscale
GCIG: Gynecological Cancer Intergroup
GCP: Good Clinical Practice
hCG: Humane Chorion Gonadotropin
HGOC: High Grade (serous) Ovarian Cancer
HR: Hazard Ratio
HRA: Federal Act on Research involving Human Beings (in German: HFG, in French: LRH, in Italian: LRUm)
ICF: Informed consent form
ICON7: International Collaborative Ovarian Neoplasm (ICON) 7 trial
IHC: Immunohistochemistry
ITT: Intention To Treat
LGOC: Low Grade (serous) Ovarian Cancer
LOGOS: Histopathological identified subgroup of patients with low-grade ovarian cancer
MATAO: Trial for maintenance therapy with aromatase inhibitor in epithelial ovarian cancer
MICD: Minimum Important Clinical Difference
OFS: Ovarian Function Suppression
OS: Overall Survival
ORR: Overall Response Rate
PARP: Poly(ADP-Ribose)-Polymerase 1
PARPi: Poly(ADP-Ribose)-Polymerase 1 inhibitor
PFS: Progression Free Survival
PD: Progressive Disease
PI: Principal Investigator
PR: Progesterone Receptor
PRO: Patient Reported Outcome
p-value: Value for probability
QoL: Health related Quality of life
QAPFS: Quality-adjusted Progression Free Survival
Q-TWiST: Quality-adjusted Time Without Symptoms and Toxicity
RCT: Randomized Clinical Trials
RECIST: Response Evaluation Criteria in Solid Tumors
RFS: Relapse Free Survival
RNA: Ribo Nucleic Acid
SAE: Serious Adverse Event
SCBR: Summary Clinical Benefit Rate
SOP: Standard Operating Procedure
TFST: Time to First Subsequent Treatment
WHO: World Health Organization
