# Supplementary material for: Maintenance Therapy with Aromatase Inhibitor in epithelial Ovarian Cancer (MATAO): study protocol of a randomized double-blinded placebo-controlled multi-center phase III Trial
Source: BMC Cancer. 2022 May 6;22:508. doi: 10.1186/s12885-022-09555-8 (PMC9074273; doi:10.1186/s12885-022-09555-8)
Supplement: Supplementary file 1 — Additional file 1: Table 1. Study schedule including procedure. [file 12885_2022_9555_MOESM1_ESM.docx]

# STUDY SCHEDULE

**Table 1** study schedule including procedure

|  | ***Screening phase*** | | | ***Maintenance treatment phase*** | | | ***Follow-up phase*** | |
| --- | --- | --- | --- | --- | --- | --- | --- | --- |
| ***Description*** | ***Pre-screening***  ***a*** | ***Screening b*** | ***Randomization***  ***c*** | ***INITIAL visit d*** | ***Study visits e*** | ***EOT***  ***(Safety visit) f*** | ***Short-term follow-up*** | ***Long-term follow-up*** |
| ***Time point***  ***M (months), W (week) / d (days) / y (years)*** | *> 1 Month before ICF-2* | *< 2 Months since last chemotherapy* | *< 2 Months since end of last chemotherapy* | *M0*  *≥ 1 Wk and ≤ 2 Wks from randomization* | *M3*  *every 3 Months for 2 yrs*  *every 6 Months for 3 yrs* | *1 Month (30 d) after last IMP* | *3 Months after last IMP* | *Every 6 Months from short-term* |
| ***Time window*** |  |  |  |  | ***+/-2 Wks*** | ***+2 Wks*** | ***+/-2 Wks*** | ***+/-2 Wks*** |
| ***Patient Information and Informed Consent g*** | ***Signed ICF-1*** | ***Signed ICF-2*** |  |  |  |  |  |  |
| ***Inclusion / Exclusion criteria*** | ***X*** | ***X*** |  |  |  |  |  |  |
| ***Randomization*** |  |  | ***X*** |  |  |  |  |  |
| ***Letrozole /Placebo dispensing*** |  |  |  | ***X*** | ***X*** |  |  |  |
| ***Demographics h*** | ***X*** |  |  |  |  |  |  |  |
| ***Medical history / Comorbidities i*** | ***X*** |  |  |  |  |  |  |  |
| ***Hematology & biochemistry j*** |  | ***X*** |  |  |  | ***X*** |  |  |
| ***Serum CA-125 k*** | ***X*** | ***X*** |  | ***X*** | ***X*** | ***X*** |  |  |
| ***Serum β-hCG l*** |  | ***X*** |  |  |  |  |  |  |
| ***Vital signs m*** |  | ***X*** |  | ***X*** | ***X*** | ***X*** |  |  |
| ***Physical & gynecological examination n*** |  | ***X*** |  | ***X*** | ***X*** | ***X*** |  |  |
| ***Performance status (ECOG) o*** | ***X*** | ***X*** |  | ***X*** | ***X*** | ***X*** |  |  |
| ***Charlson Comorbidity Index (age-adjusted: AACCI) p*** | ***X*** |  |  |  |  |  |  |  |
| ***G8 Geriatric Score p, q*** | ***X*** |  |  |  |  |  |  |  |

| ***ESGO Surgery questionnaire p, r*** | ***X*** |  |  |  |  |  |  |  |
| --- | --- | --- | --- | --- | --- | --- | --- | --- |
| ***Cancer characterization s*** | ***X*** | ***X*** |  |  |  | ***X***  *(only response)* |  |  |
| ***Paraffin block t*** | ***X*** |  |  |  |  | ***At progression***  *(if available)* |  |  |
| ***Routine Imaging u*** |  | ***X*** |  |  |  | ***X*** |  |  |
| ***Resilience v*** |  | ***X*** |  |  |  | ***X*** |  |  |
| ***QoL Questionnaires w, y*** |  |  |  | ***X*** | ***X*** | ***X*** | ***X*** |  |
| ***Activity tracker x, y*** |  | ***Hand out*** |  | ***X*** | ***X*** | ***X*** |  |  |
| ***Survival Status z*** |  |  |  | ***X*** | ***X*** | ***X*** | ***X*** | ***X*** |
| ***Concomitant medication*** | ***X*** | ***X*** |  | ***X*** | ***X*** | ***X*** |  |  |
| ***Adverse events*** |  |  |  | ***X*** | ***X*** | ***X*** |  |  |


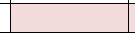
 Activities specific for study conduct.

1. The pre-screening visit will take place > 1 month before screening visit (since ER measurement and histopathological review will take 1 month).
2. Screening visit: < 2 months after last platinum-based chemotherapy cycle, after centralized pathology review and positive ER-expression testing (IHC) / biobanking.
3. Randomization: < 2 months from last platinum-based chemotherapy cycle. When all inclusion and none of the exclusion criteria are met, then randomization is done.
4. Initial visit (M0): ≥ 1 week and ≤ 2 weeks from randomization.
5. First visit after 3 months (M3) from initial visit (M0) and then every 3 months for 2 years. After two years, the visit interval will change to 6 months for another 3 years according to scheduled routine visits.
6. EOT (end of trial visit): this visit will be scheduled 1 month (+ 2 weeks) from last IMP administration (it is also known as safety visit, see 9.3.7).
7. Potential study candidates will be informed about the MATAO study prior to the pre-screening visit.
8. Demographic data includes date of birth, BMI (height, weight), etc.
9. Medical history / comorbidities includes: BRCA mutation status (if available), surgeries, malignancies, family history for malignancies, hypertension, and diabetes. To be updated throughout the study.
10. Hematology (erythrocytes, hemoglobin, leucocytes, and thrombocytes) and biochemistry (bilirubin, ASAT (Aspartate Aminotransferase)), ALAT (Alanine Aminotransferase), AP (Alkaline Phosphatase), creatinine, sodium, potassium, calcium, magnesium, chloride and vitamin D) analysis within routine laboratory practice.
11. Serum CA-125 within routine laboratory practice. Additional CA-125 testing may be performed according to investigator’s discretion.
12. Serum β-hCG: if positive, potential patient is ineligible for study enrolment.
13. Vital signs: blood pressure, heart rate, temperature
14. Physical & gynecological examination: Includes nodal status (Virchow groins), auscultation of lungs, examination of abdomen, liver, genital inspection and palpation.
15. ECOG performance status scores are provided in annex.
16. ESGO surgery, Age Adjusted Charlson Comorbidity Index (AACCI), G8 score if these questionnaires were assessed before the surgery as routine procedures, patients under chemotherapy are allowed to be considered for study participation.
17. Geriatric score: for patients ≥ 75 year.
18. ESGO surgery questionnaire: to be recorded at initial debulking or interval debulking.
19. Ovarian cancer characterization: Date of ovarian cancer diagnosis & follow-up, FIGO stage, subtype, grade, response.
20. Tumor/cell paraffin block - after primary diagnosis and surgery: a coded representative biopsy block conserved with paraffin to be sent to Histopathology Core Facility of the MATAO Trial. The trial site sending the block will get the result of the ER measurement and histopathological review within 1 month.
21. Imaging: according to local standard of care procedures, not required for trial conduct, but if performed RECIST v1.1 is recommended.
22. Resilience will be assessed by the CD-RISC-10 questionnaire, has to be completed by the patient on paper (not electronically!)
23. QoL questionnaires: include EQ-5D-5L, FACT-ES and FACT-O digitally.
24. Activity Tracker: Patients will receive an activity measurement device to wear at the arm for 1 week before their consultation. If patient shows a progressive disease after platinum-based chemotherapy and therefore do not meet the eligibility criteria, then the patient shall return activity tracker at next clinical visit.
25. QoL questionnaires & activity tracker: study nurse should remind patient to wear the activity tracker and to complete the questionnaire App (if applicable) approximately 8 days before each visit
26. Survival status: up to 5 years (MATAO, low & high grade) and up to 7 years (only low grade) after the last randomized patient.

**Note:** Unscheduled visits can occur at any time. Procedures to be performed as indicated. Depending on the result of examination, they can lead into resumption of regular study visits or EOT. (S)AEs to be documented and reported as required.

**Note 1:** There will be two ICFs for the study:

- - By signing the first ICF (ICF-1) the patient gives permission to use a sample of her tumor tissue for a centralized pathology review, ER expression testing (IHC) and biobanking. The paraffin-block has to be sent to the centralized Histopathology Core Facility of the MATAO Trial and the trial site sending the block will get the result of IHC within 1 month. The first ICF will be obtained from the patient by the responsible site before the end of the systemic therapy.
  - The second ICF (ICF-2) will be obtained from the patient by the responsible site < 2 months from the end of the last platinum-based chemotherapy cycle.

**Note 2:** Once progression is identified, subsequent treatment (systemic, surgery, radiation) information shall be documented as this information and data is important in relation to overall survival.
