# Supplementary material for: Maintenance Therapy with Aromatase Inhibitor in epithelial Ovarian Cancer (MATAO): study protocol of a randomized double-blinded placebo-controlled multi-center phase III Trial
Source: BMC Cancer. 2022 May 6;22:508. doi: 10.1186/s12885-022-09555-8 (PMC9074273; doi:10.1186/s12885-022-09555-8)
Supplement: Supplementary file 2 — Additional file 2: Ethical committees & Study sites ENGOT-ov54/Swiss-GO-2/MATAO(LOGOS). [file 12885_2022_9555_MOESM2_ESM.docx]

**Ethical committees & Study sites ENGOT-ov54/Swiss-GO-2/MATAO(LOGOS)**

**Switzerland**

ENGOT-ov54/Swiss-GO-2/MATAO

*Lead Ethical committee:*

The Ehikkommission Nordwest-Zentralschweiz (EKNZ): Chair; Prof Christoph Beglinger, Deputies; Dr Angela Frotzier, Dr Marco Schärer. Hebelstrasse 53, 4056 Basel, Tel +41 61 268 1350, [eknz@bs.ch](mailto:eknz@bs.ch), [www.eknz.ch](http://www.eknz.ch); Project-ID: 2020-00148

*Ethical committees involved:*

- Ethikkommission Nordwest- und Zentraischweiz (EKNZ)
- Kantonale Ethikkommission Bern (BE)
- Ethics Committee Geneva (GE)
- Ethikkommission Ostschweiz (EKOS)
- Ethikkommission Tessin (TI)
- Ethics Committee Vaud (VD)
- Ethikkommission Zürich (ZH)

*Study sites:*

1. Unispital Basel (USB), 2. Kantonsspital Aarau (KSA), 3. Kantonsspital Baden (KSB),

4. Kantonsspital Graubünden-Chur (KSGR), 5. Stadtspital Triemli Zürich (STZ),

6. Bern Lindenhofgruppe, 7. Luzern Kantonsspital (LUKS), 8. Zürich Onkozentrum Hirslanden (OnkoZ), 9. Lausanne (CHUV), 10. Basel Claraspital, 11. Kantonsspital St. Gallen (KSSG)

12. Hôpitaux universitaires Genève (HUGE), 13. Thurgau AG-KS Frauenfeld (TG-KSF)

14. Spital Grabs (SRRWS), 15. Kantonsspital Winterthur (KSW), 16. Bern Inselspital ,17. Thurgau AG-Münsterlingen (TG-KSM), 18. Unispital Zürich (USZ), 19. KS Baselland Liestal (KSBL)

20. Hirslanden Klinik St. Anna Luzern (KLSA),21. Instituto Oncologico Svizzera Italiana Bellinzona (IOSI )

**Austria**

ENGOT-ov54/Swiss-GO-2/AGO 65/MATAO(LOGOS),

*Lead Ethical committee:*

Ethikkommission der Medizinische Universität Innsbruck, Innrain 43 A-6020 Innsbruck; [ethikkommission@i-med.ac.at](mailto:ethikkommission@i-med.ac.at); [www.i-med.ac.at/ethikkommission](http://www.i-med.ac.at/ethikkommission)

EK Ref Nr: EK 1496/2020

*Ethical committees involved:*

- Ethikkommission der Medizinischen Universität Innsbruck
- Ethikkommission der Medizinischen Universität Graz
- Ethikkommission der Medizinischen Universität Wien
- Ethikkommission der Medizinischen Fakultät der JKU
- Ethikkommission für das Bundesland Salzburg

*Study sites:*

1. Medizinische Universität Wien (MUW), 2. Klinik Hietzing Wien Hietzing (KHWH), 3. Medizinische Universität Graz (MUG),4. Krankenhaus Barmherzige Brüder Graz (KHBBG), 5. Krankenhaus Barmherzige Schwestern Linz (KH BHS), 6. Medizinische Universität Innsbruck (MUI)

7. Landeskrankenhaus Hochsteiermark Leoben (LKHH), 8. Universitätsklinikum Salzburg (SALK)

**Germany**

ENGOT-ov54/Swiss-GO-2/AGO-OVAR26/MATAO(LOGOS)

*Lead Ethical committee:*

Albert-Ludwigs-Universität Freiburg Ethik-Kommission; Prof. Dr. R. Korinthenberg (Vorsitzender); Engelberger Straße 21◦ 79106 Freiburg; Tel. 0761/270-72600 / -72500; ekfr.amg@uniklinik-freiburg.de; [www.ethik-kommission.uniklinikfreiburg.de](http://www.ethik-kommission.uniklinikfreiburg.de); Antrags-Nr.: 22-1004-AMG-ff multi.

*Ethical committees involved:*

- Ethikkommission der Universität Ulm
- Ethikkommission II, Medizinische Fakultät Mannheim der Universität Heidelberg
- Ethikkommission der LÄK Baden-Württemberg
- Ethikkommission an der Universität Regensburg
- Ethikkommission der Bayerischen Landesärztekammer
- Ethikkommission der Fakultät für Medizin der Technischen Universität München
- Ethikkommission der Medizinischen Fakultät der Ludwig-Maximilians Universität, München
- LaGeSo Berlin, Geschäftsstelle der Ethik-Kommission des Landes Berlin
- Ethikkommission der ÄK Hamburg
- Ethikkommission der LÄK Hessen
- Ethikkommission bei der ÄK Niedersachsen
- Ethikkommission an der Medizinischen Fakultät der Heinrich-Heine-Universität Düsseldorf
- Ethikkommission der ÄK Nordrhein
- Ethikkommission der ÄK Westfalen-Lippe und der Westfälischen Wilhelms-Universität Münster
- Ethikkommission der Landesärztekammer Rheinland-Pfalz
- Ethikkommission an der Technischen Universität Dresden
- Ethikkommission der Medizinischen Fakultät der Christian-Albrechts-Universität zu Kiel

*Study sites:*

1. Universitätsklinikum Freiburg, 2. Universitätsklinikum Münster, 3. AMO Wolfsburg / AMO MVZ GmbH, 4. LMU Klinikum der Universität München, 5.Kliniken der Stadt und des Landes Rosenheim GmbH, 6. Universitätsklinik Ulm; 7.Universitätsklinikum Düsseldorf, 8.Städtisches Klinikum Solingen, 9. Universitätsklinikum Carl Gustav Carus Dresden an der Technischen Universität Dresden, 10. St. Elisabeth-Krankenhaus GmbH, 11. AGAPLESION Markus Krankenhaus

12. Universitätsklinikum Mannheim GmbH, 13. Universitätsklinikum Schleswig-Holstein

14. Universitätsklinikum Hamburg-Eppendorf, 15. Charité Universitätsmedizin Berlin Campus Virchow Klinikum, 16. Helios Dr. Horst Schmidt Kliniken Wiesbaden GmbH, 17. Klinikum Hochsauerland GmbH, Karolinen-Hospital, 18. Donauisar Klinikum, 19. Universtitätsmedizin Mainz

20. Gynäkologisch-Onkologische Gemeinschaftspraxis Dres. med. C.Uleer/J.Y.Pourfard, 21. Klinik für Frauenheilkunde und Geburtshilfe der Universität Regensburg am Caritas-Krankenhaus, 22. Leopoldina-Krankenhaus der Stadt Schweinfurt, 23. Studienzentrum Onkologie Ravensburg

24.Evang. Kliniken Essen-Mitte, 25. Klinikum Konstanz, 26. Vivantes Klinikum am Urban.
